# Microstimulation of single human motor axons in the toe extensors: force production during long‐lasting trains of irregular and regular stimuli

**DOI:** 10.14814/phy2.13067

**Published:** 2017-02-27

**Authors:** Michael Leitch, Vaughan G. Macefield

**Affiliations:** ^1^ School of MedicineWestern Sydney UniversitySydneyAustralia

**Keywords:** Discharge variability, force generation, microstimulation, single axons

## Abstract

Human motoneurones are known to discharge with a physiological variability of ~25% during voluntary contractions. Using microstimulation of single human motor axons, we have previously shown that delivering brief trains (10 pulses) of irregular stimuli, which incorporate discharge variability, generates greater contractile responses than trains of regular stimuli with identical mean frequency but zero variability. We tested the hypothesis that longer irregular (physiological) trains would produce greater contractile responses than regular (nonphysiological) trains of the same mean frequency (18 Hz) and duration (45 sec). Tungsten microelectrodes were inserted into the common peroneal nerve of human subjects, and single motor axons supplying the toe extensors (*n* = 14) were isolated. Irregular trains of stimuli showed greater contractile responses over identical mean frequencies in both fatigue‐resistant and fatigable motor units, but because the forces were higher the rate of decline was higher. Nevertheless, forces produced by the irregular trains were significantly higher than those produced by the regular trains. We conclude that discharge irregularity augments force production during long as well as short trains of stimulation.

## Introduction

Human motoneurones are known to exhibit significant discharge variability during voluntary contractions, which is known to fall as mean firing rate increases (Stålberg and Thiele 1973; Moritz et al. 2004; Laidlaw et al. 2000). Physiological discharge variability includes short and long interspike intervals and it has been shown that incorporating a short interspike interval (‘doublet’) at the beginning of a train of electrical stimuli augments the contractile response. This has been established in animal models (Callister et al. 2003) as well as in humans – both during stimulation of whole human muscle (Binder‐Macleod and Lee 1996) and during microstimulation of single human motor axons (Macefield et al. 1996).

Intraneural microstimulation allows the twitch properties and force‐frequency responses of individual motor units to be examined, with no bias toward low‐ or high‐threshold motor units (Macefield et al. 1996; Fugelvand et al. 1999; McNulty et al. 2000; Thomas et al. 1991a; Westling et al. 1990). Until recently, however, all studies using intraneural microstimulation of human motor axons have measured the contractile responses to trains of regular stimuli, but we wanted to determine the influence of physiological discharge variability on the response of individual motor units. In an earlier study we stimulated single motor axons supplying the long toe‐extensor muscles with short trains (10 pulses) of regular and irregular stimuli. Two conclusions were drawn from this study. Firstly, contractile responses (measured as angular displacements of the toe) were higher for irregular trains than regular trains over identical mean frequencies (8–24 Hz). Secondly, when the motor unit was then subjected to a 2‐min train of stimuli that led to a reduced amplitude of the evoked force, subsequent delivery of the short irregular trains resulted in significantly higher contractile responses than did the regular trains (Leitch and Macefield 2014). The continuous train was given as a regular set of stimuli at 10 Hz, so we do not know how motor units behave during a continuous train of *irregular* stimuli. It was of interest, therefore, to test whether the same augmentation of contractile response could be exhibited when incorporating physiological discharge variability into longer trains of stimuli that closely resemble the static isometric contractions performed voluntarily. The purpose of this study was to test this hypothesis, by delivering trains of stimuli (18 Hz for 45 sec) with and without discharge variability and measuring force production by individual motor units in the long toe‐extensor muscles.

### Functional implications

Functional electrical stimulation (FES) has shown great potential as a rehabilitation therapy after injuries that affect the human motor system. It has been used after stroke and spinal cord injury (SCI) to assist in ambulation and to improve reach‐to‐grasp tasks (Miller et al. 1990; Billian and Gorman 1992). It has also been used in stimulation of the peroneal nerve to treat foot drop (Waters et al. 1975; Voigt and Sinkjaer 2000) and to help in bladder control of paraplegics (Brindley 1973; Jonas et al. 2001). FES of whole muscle, delivered via surface electrodes, has been shown to increase muscle mass, improve contractility, and increase force output (Scremin et al. 1999; Baker et al. 1979).

The current patterns of stimulation applied in this therapy utilize constant‐current, high‐frequency, stimulation trains – typically ranging from 30–100 Hz. One of the major limitations with this therapy is muscle fatigue. It is well known that stimulating with high‐frequency trains causes rapid fatigue (Bigland‐Ritchie et al. 1979; Garland et al. 1988) and, thus, the long‐term efficacy for rehabilitation purposes may be underestimated. Because SCI patients have muscles predominately composed of fast‐fatigable muscle fibers then this could be a reasonable explanation for the rapid fatigue exhibited in these patients during FES therapy (Burnham et al. 1997). However, another concern is the order in which the motor units are activated. Henneman's size principle states that the smallest motor units supplying the least number of fibers are recruited first (owing to their higher input resistance), through to the largest ones, supplying the most fibers, last (Henneman et al. 1965a,b; Olson et al. 1968). This results in slow ramp voluntary contractions being graded in a linear fashion (Milner‐Brown et al. 1973; Bigland and Lippold 1954). However, the smallest motoneurones have the smallest diameter axons, and hence have the highest electrical thresholds to direct electrical stimulation, whereas the largest motoneurones – with larger axons – have the lowest electrical thresholds. This means that when stimulating whole muscle, the largest motor axons will be preferentially activated, and these will be the most susceptible to fatigue. It is only through supramaximal stimulation that all motor axons will be excited and small, fatigue‐resistant, motor units recruited. Conversely, microstimulation of motor axons via an intraneural microelectrode does not favor large over small axons: the axon closest to the tip of the stimulating microelectrode is the one that is recruited at the lowest currents, and we know from our own work, and that of others, that the whole range of twitch types is sampled essentially randomly.

## Methods

Experiments were conducted on 11 male and 2 female subjects (18–36 years) under the approval of the Human Ethics Committee of Western Sydney University. Subjects provided informed written consent. Successful microstimulation of 14 single motor axons in extensor digitorum longus (EDL) and extensor hallucis longus (EHL) was performed in nine subjects.

The participants were reclined in a chair with the knee flexed to approximately 120^o^. The foot was fixed onto a rigid footplate with the ankle at 120^o.^ A Velcro strap was attached over the dorsum of the foot, proximal to the digits (so as not to interfere with dorsiflexion of the toes), to anchor the foot to the footplate. The leg was supported by a vacuum cast to prevent any movement during the procedure. The common peroneal nerve was located using a weak electrical stimulus (0.02–10.00 mA, 0.2 msec, 1 Hz; Stimulus Isolator, ADInstruments, Sydney, Australia) delivered by a 2‐mm‐diameter surface probe. A high‐impedance tungsten microelectrode (Frederick Haer & Co, Bowdoin, ME), 200 *μ*m in diameter, was inserted into the nerve and an Ag/AgCl surface electrode on the opposite side of the knee served as the anode. Constant‐current cathodal pulses (0.02–1.00 mA, 0.2 msec, 1 Hz) were delivered through the microelectrode and the microelectrode was manually adjusted to search for a motor fascicle supplying either EHL or EDL. EMG was recorded with surface Ag/AgCl electrodes placed over tibialis anterior, fibularis longus/brevis, and extensor digitorum brevis, amplified (BioAmplifier, ADInstruments, Sydney) and filtered (10 Hz–1 kHz) and recorded (2 kHz sampling) on a computer‐based data acquisition and analysis system (PowerLab 16 SP hardware and LabChart Pro 7 software, ADInstruments, Sydney). Force (DC‐100 Hz; 200 Hz sampling) was measured using a highly sensitive angular force transducer (Model 1030; UFI, Morro Bay, CA) located over the distal phalanx of the stimulated toe – either the big toe for EHL or the second or third toe for EDL.

A single motor axon was activated when microstimulation generated a small twitch and a reproducible EMG potential at the lowest possible current within a well‐defined safety margin, typically 3–7 *μ*A, in which increases in current failed to modify the twitch amplitude or EMG profile over the defined safety margin. The motor axon was then stimulated with sets of 8 pulses at the threshold current in order to measure the twitch properties of the motor unit. Twitch parameters were measured using the Peak Parameters feature of LabChart. After establishing that a single motor axon had been isolated trains of 10 regular pulses from 2–18 Hz were delivered to construct a force‐frequency curve. Following these trains, an irregular (physiological) long‐lasting train (18 Hz, ~800 spikes) was given for 45 sec; this allowed us to create a time‐varying picture of the contractile responses generated by the long‐lasting trains on active motor units. The subject then had sufficient recovery time to allow contractile responses to return to peak threshold. This could take up to 15 min depending on the type of motor unit stimulated (fatigable or fatigue resistant). We intermittently gave the 10‐pulse trains (2–18 Hz) in the same manner as with the irregular train. This allowed us to monitor when contractile responses had returned to normal. Once the forces were identical to those delivered before the irregular long‐lasting train, the regular (constant frequency) long train was delivered – with the same mean frequency, yet zero variability.

### Generating the irregular long‐lasting trains

The irregular long‐lasting train was based on the discharge variability in single motor units recorded from tibialis anterior during a series of voluntary isometric contractions. For this purpose a tungsten microelectrode was inserted directly into the muscle belly of tibialis anterior and amplified (gain 1000×, 300 Hz–5 kHz; 10 kHz sampling; NeuroAmp EX, ADInstruments, Sydney). Spike trains from individual motor units or individual muscle fibers were recorded during dorsiflexion at the ankle and the microelectrode adjusted so as to obtain several spike trains of several seconds in duration within a given contraction. The subject was asked to sustain a ~30% maximal voluntary contraction for 45 sec. Firing rates ranged over 145.83 with a maximum of 151.52 and minimum of 5.69. The mean frequency for this train was 18.3 ± 8.7 with discharge variability of ~25.3% (SD/mean × 100) based on the coefficient of variation. The interspike intervals exhibited by this train were used to construct an irregular (physiological) long‐lasting train lasting for 45 sec in duration.

### Regular long‐lasting trains

The long‐lasting regular train consisted of the same number of pulses as the irregular train. This meant that we had an identical number of spikes in both trains and an identical mean frequency, but with one train without and one train with discharge variability. This allowed us to determine whether greater responses are seen when incorporating physiological variability. All statistical analyses were performed using Prism 6 software (GraphPad Software, San Diego, CA).

## Results

### Motor unit characteristics

**Figure 1:**
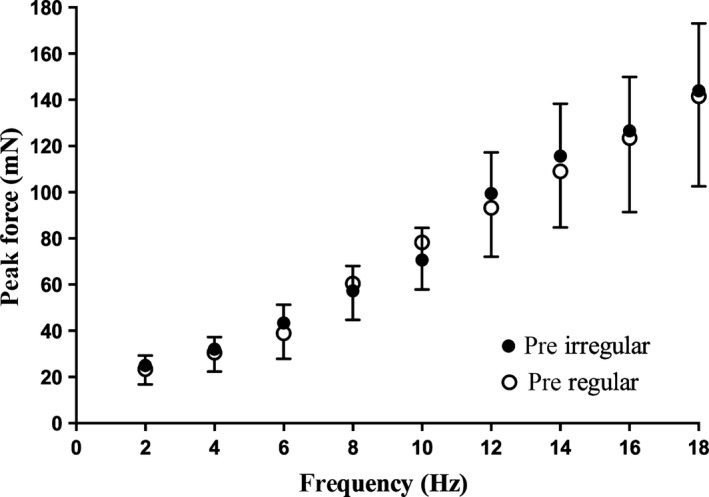
Force‐frequency curves for all 14 motor units, delivered as short trains of 10 pulses at frequencies of 2–18 Hz, before delivery of the long trains of either irregular or regular stimuli. There were no significant differences in peak force (± SE) at any frequency.

Successful microstimulation of a single motor axon was performed on 14 motor units supplying the long toe extensors. Once a single motor axon was isolated, twitch properties measured, and a force‐frequency curve generated, a 45‐sec train of irregular stimuli was delivered at 18 Hz. This was followed by the generation of another set of pulses to measure twitch properties and another force‐frequency curve to assess whether the unit had recovered from any decline in force. After a variable recovery time this was repeated (up to three times) until the twitch properties and force‐frequency relationships had normalized. Five of the 14 units were deemed fatigable units, based on their relatively long recovery time, whereas the other 9 units recovered very rapidly. Therefore, the second (regular) long‐lasting train could be delivered almost immediately for these units, given that the twitch properties and force‐frequency curve had not changed from baseline values. Figure 1 shows that the force‐frequency curves generated before the irregular or regular continuous train; there were no significant differences between the curves at any frequency, indicating that the motor unit properties had normalized before the regular long train was given.

### Effects of discharge variability on force production

**Figure 2:**
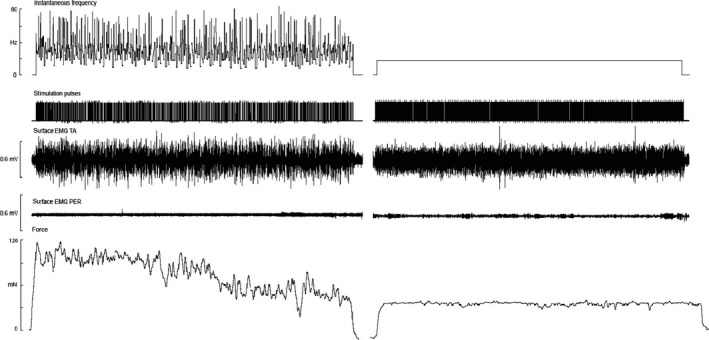
Experimental records from one subject. Microstimulation of a single motor axon supplying extensor digitorum longus (EDL) with a 45‐sec train, delivered at a mean frequency of 18 Hz with (left panel) and without (right panel) variability. Channels 1 and 2 show the instantaneous frequency and corresponding stimulation pulses for both the irregular and continuous (regular) frequency trains. It can be seen that the irregular train exhibits significant discharge variability over the 45 sec compared with the regular train (zero variability). The top EMG channel shows significant electromyographic activity from surface electrodes over the anterior leg (TA), due to activation of a single motor axon in an EDL fascicle of the anterior compartment. The bottom EMG channel shows zero EMG activity over the lateral peroneus muscles (PER). The bottom channel displays the force responses generated by the two types of trains (irregular and regular). The irregular trains generate significantly greater force responses over the entirety of the 45‐sec train. This was confirmed in all subjects (*n* = 14).

**Figure 3:**
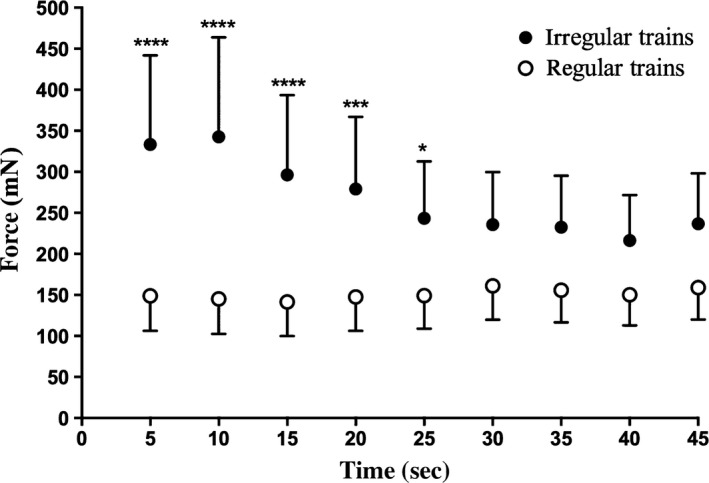
Peak force generated by 14 single motor units, comparing contractile responses produced by irregular and regular long‐lasting trains of stimuli. Forces generated by the irregular stimulus trains were significantly higher than those generated by the regular trains in the first 25 sec. ****P < 0.0001; *P < 0.05.

Experimental records from one motor unit are shown in Figure 2. It can be seen that the irregular trains generated a much higher force at the beginning of the contraction, yet this declined in the last 20 sec. Figure 3 shows the time courses of the force generated during microstimulation of all 14 single motor axons with long trains of irregular or regular stimuli. The overall mean difference in force generated between the irregular and regular trains was 184.3 ± 11.5 mN in the first 5 sec and 81.3 ± 22.4 mN at the conclusion of the train (45 sec). So, while there was evidence of a decline in force during the course of delivery of the irregular trains, the regular trains produced a very stable force throughout the 45 sec for all 14 single motor axons. However, the regular trains never generated forces that exceeded those produced by the irregular trains. As shown in Figure 3, the forces generated by the irregular trains were significantly greater over the first 20 sec (*P* < 0.0001), but not over of the last 20 sec.

**Figure 4:**
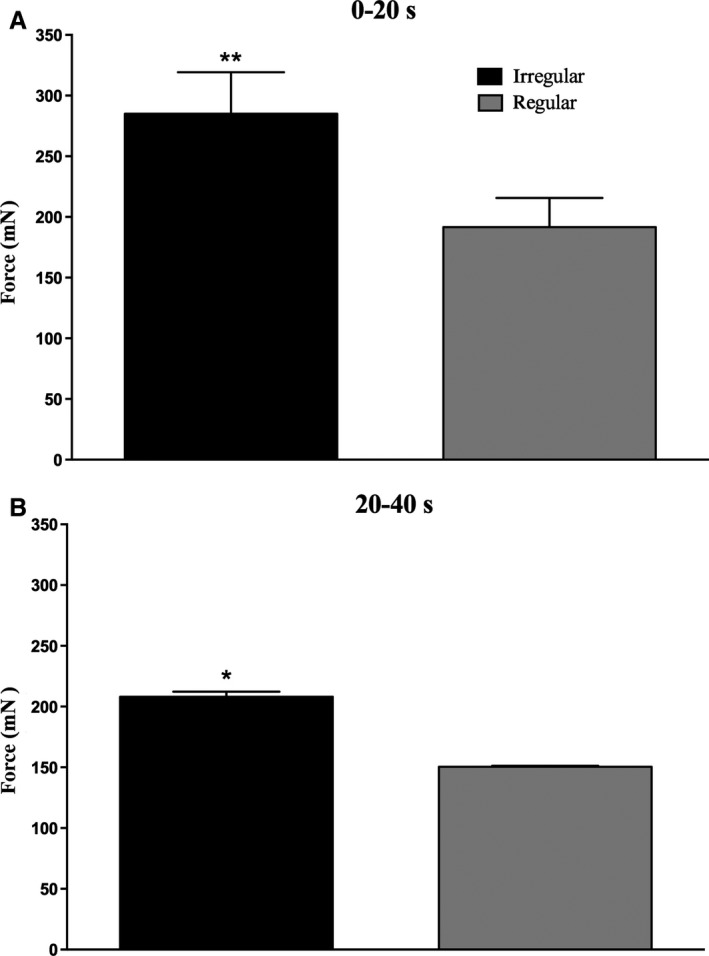
Total mean forces generated by 14 single motor axons. Mean forces generated during the first 20 sec (A) and the last 20 sec (B) were significantly greater for the irregular trains. **P < 0.01; *P < 0.05.

Fig. 4 shows the total mean forces generated, when pooled over the first 20 sec and over the last 20 sec, for both the irregular and regular trains of stimuli. Mean forces, computed over 20 sec, were significantly higher in both phases for the irregular than the regular trains, delivered with an identical mean frequency (18 Hz).

## Discussion

The purpose of this study was to test the hypothesis that incorporating discharge variability into a long‐lasting train of stimuli leads to augmented force production. In other words, the physiological discharge variability seen in the firing of single motor units during voluntary contractions offers advantages to the neuromuscular system. Our previous study showed that when incorporating physiological variability into short 10‐pulse trains of stimuli there is an increase in peak contractile response, over mean frequencies ranging from 8–24 Hz (Leitch and Macefield 2014). This finding was the catalyst for further exploration into the potential advantages offered by incorporating physiological discharge variability into a train of stimuli. We have shown that the addition of physiological variability into long‐lasting trains produces greater forces, but that this declines over time. Presumably, this decline in force production reflects the fact that forces were higher at the onset of the contraction produced by an irregular train, despite the mean frequency being identical to that of the regular train. In other words, the muscle itself cannot sustain the higher force generated by a train of irregular stimuli delivered at a mean frequency of 18 Hz. Nevertheless, given that the stimulation patterns we have generated emulate the firing of *α* motoneurones during voluntary contractions, one could expect that the nervous system utilizes discharge variability as a method of increasing force production without increasing the firing rate.

### Methodological considerations

One of the fundamental challenges in isolating a single motor unit is to selectively stimulate one and only one motor axon within a muscle fascicle of the nerve. This requires very low currents and meticulous care to direct the tip of the microelectrode into a fascicle supplying one of the toe‐extensor muscles. Very high impedance tungsten electrodes were used, and careful monitoring of the twitch profiles and EMG potential was crucial. We were convinced that we had isolated a single motor axon when the EMG and twitch potentials were reproducible at the lowest possible current, with a safety margin usually between 3–7 *μ*A. Within the safety margin any increases in current failed to increase the twitch and EMG of the active unit. Therefore, stimulating within this safety margin did not result in the recruitment of any additional motor axons. The trains of stimuli were delivered in the same order each time. Firstly, the irregular long‐lasting train was delivered, followed by the regular long‐lasting train – with sufficient recovery time between the two trains. One way to prevent any bias between the two types of trains would have been to randomize the order in which the trains were given. However, we carefully monitored the contractile responses during recovery by repeating a force‐frequency curve (10 pulses, 2–18 Hz) until forces had normalized. This ensured that the muscle had fully recovered from any short‐term decline in force and therefore the order in which the trains were given did not need to be randomized.

### Mechanistic considerations

Animal work suggests that the increased contractile responses to stimulation with irregular trains could be explained purely on a biomechanical basis. Because muscle contractions are sluggish, they act as low‐pass filters that limit the conversion of a motor unit action potential delivered by a motor axon into a contractile response. Accordingly, when a second stimulus is given before the motor unit has fully relaxed from the preceding stimulus, it prevents relaxation and produces augmentation of force, known as a catch‐like property. This phenomenon was first observed in animals, in slow‐twitch motor units in the triceps surae muscles of the cat (Burke et al. 1970) and has also been shown to produce greater tetanic responses in single motor units of the rat medial gastrocnemius (Celichowski and Grottel 1998). One possible explanation for this natural occurrence relates to calcium reuptake. When an action potential is propagated along the sarcolemma to the transverse tubule, a signal from the t‐system initiates calcium release from the sarcoplasmic reticulum into the myoplasm; if an additional stimulus were given before calcium reuptake occurred, then a decline in force would be seen.

It has been shown that calcium sensitivity of contractile proteins is unchanged during low‐frequency fatigue (Westerblad et al. 1993) and that the likely cause of low‐frequency fatigue is due to lack of calcium release from the sarcoplasmic reticulum (Westerblad et al., 1993). Therefore, a possible mechanism with regard to this research may be that calcium does not have a chance to be released as efficiently during long‐lasting physiological trains due to slower reuptake of calcium caused by the shorter interspike intervals inherent in an irregular stimulus train. It is therefore not unreasonable to suggest that irregular stimuli would result in greater forces at the beginning of a long train, yet result in greater rate of decline in force toward the end of the train, due to lack of available calcium. Irregular trains showed a greater rate of decline throughout the 45 sec, but at the end of the train the force was still higher than that seen with the regular train. Previous studies have shown that initial doublets (10‐msec interpulse interval) at the onset of stimulus train yielded an increased contractile response, both in whole muscle (Binder‐Macleod and Barker 1991 Binder‐Macleod and Lee 1996; Lee et al. 1999) and in single motor units (Macefield et al. 1996). However, until now there have been no studies that have assessed the effects of adding physiological variability to a long‐lasting train.

## Conclusions

We have shown that incorporating physiological variability into long‐lasting trains of stimuli offers an advantage to the neuromuscular system. Irregular trains showed significantly greater contractile responses for the first 20 sec of the train with a greater rate of decline than regular trains over the same mean frequencies (18 Hz, 45 sec). However, while irregular long‐lasting trains showed a greater rate of decline the force never dropped below that produced by a regular train of identical mean frequency with zero variability.

## Conflict of Interest

The authors declare no conflict of interest.
